# Deceiving scientific research, misconduct events are possibly a more common practice than foreseen

**DOI:** 10.1186/s12302-022-00659-3

**Published:** 2022-08-23

**Authors:** Alonzo Alfaro-Núñez

**Affiliations:** 1 Department of Clinical Biochemistry, Naestved Hospital, Ringstedgade 57a, 4700 Naestved, Denmark; 2 Section for Evolutionary Genomics, GLOBE Institute, University of Copenhagen, Øster Farimagsgade 5, 1353 Copenhagen K, Denmark

**Keywords:** Ethical values, Transparency, Plagiarism, Scientific fraud, Research misconduct and respect

## Abstract

**Background:**

Today, scientists and academic researchers experience an enormous pressure to publish innovative and ground-breaking results in prestigious journals. This pressure may blight the general view concept of how scientific research needs to be done in terms of the general rules of transparency; duplication of data, and co-authorship rights might be compromised. As such, misconduct acts may occur more frequently than foreseen, as frequently these experiences are not openly shared or discussed among researchers.

**Main body:**

While there are some concerns about the health and the transparency implications of such normalised pressure practices imposed on researchers in scientific research, there is a general acceptance that researchers must take and accept it in order to survive in the competitive world of science. This is even more the case for junior and mid-senior researchers who have recently started their adventure into the universe of independent researchers. Only the slightest fraction manages to endure, after many years of furious and cruel rivalry, to obtain a long-term, and even less probable, permanent position. There is an evil circle; excellent records of good publications are needed in order to obtain research funding, but how to produce pioneering research during these first years without funding? Many may argue this is a necessary process to ensure good quality scientific investigation, possibly, but perseverance and resilience may not be the only values needed when rejection is received consecutively for years.

**Conclusion:**

There is a general culture that scientists rarely share previous bad experiences, in particular if they were associated to misconduct, as they may not be seen or considered as a relevant or hot topic to the scientific community readers. On next, a recent misconduct experience is shared, and a few additional reflections and suggestions on this topic were drafted in the hope other researchers might be spared unnecessary and unpleasant times.

## Background

Scientists are under great pressure to publish not only high-quality research, but also a larger number of publications, the more the merrier, within the first years of career in order to survive in the competitive world of science. This pressure might mislead young less experienced researchers to take “shortcuts” that may consequently mislead to carry out misconduct actions. The aim of this article is not just trying to report a case of misconduct to the concerned stakeholders, but also to the research community as a whole in the hope other researchers might avoid similar experiences. Moreover, some basic recommendations are shared to remind the basic rules of transparency, duplication of data and authorship rights to avoid and prevent misconduct acts based on existing literature and the present experience.

## Welcoming collaboration

During the first months of 2021, already in the second year of the COVID-19 pandemic with most European research institutes and labs still in lockdown [1], and all over the world, I received an email from a young researcher overseas. This young fellow is based in Bangladesh, South Asia, in a country in which I have never collaborated before. He was interested in a potential collaboration with many ideas, and proved to be a very energetic person writing me on a daily basis and even several times a day during the first weeks.

There were obviously some suspicions about the nature of this collaboration, but the general and basic background check out was done, and this fellow seemed to be legitimate. Thus, after a few weeks of discussing back and forth research ideas, I welcomed the collaboration. Thereafter, for the first few months many ideas were elaborated and discussed, and so we began to draft two review manuscripts simultaneously. In no time, it felt like a potential and long-standing collaboration was born. However, it also required additional time because of the linguistic and cultural barrier. It appeared that sometimes the main message was getting lost in translation, and it was reflected in the text on the various manuscript versions. We repetitively argued about the importance of transparency, the correct use of data previously published and the general rules of authorship and citation, especially when producing a new review document. Nevertheless, these errors were corrected and he guaranteed to have full understanding, and I trusted.

After some time, enthusiasm started to decline and the highly motivated collaborator started to rush to complete the work regardless of the quality, especially as a third manuscript was now also in play. I was not willing to sacrifice quality, so I started using more of my personal time to complete the different manuscripts, I felt committed. After six months or so, the first of the three manuscripts was ready, and the process of submission started to a high-impact peer-review journal to a special issue on a topic where I had been invited months ago. A few months later, the second manuscript followed the same steps.

By the middle of April 2022, the first of the manuscripts had just been accepted; the second one was already in its second round of review, and the third and last of the manuscripts was ready for submission. I cannot deny the satisfaction felt of a good job properly done in a time record (for my personal standards).

## Deceptive surprise

Through the last hours, before submitting our final manuscript, the mandatory final inspection was done. However, I noticed something odd, two new citations had been added in the last minute, and I did not approve that change. Even more curious, the two citations had the new collaborator’s name on it. Immediately, I searched for the two mysterious documents, a book chapter and another peer-reviewed publication were the result. To my surprise, the titles of these two new works were very similar and somehow nearly identical to the topic we had just finished and his name appeared as the first author. Both documents were not open access and had recently been published, one of them less than a week old. Furthermore, our manuscript, the same document I was supposed to submit that same day, had six figures and four tables, all generated by our collaborative work. The book chapter had exactly the same figures and tables just in a different order, but the data and content were nearly identical. The text redaction was different, and there were also some other co-authors from his same region, but the content and background idea was the same.

During the next hours, I went back to the other two manuscripts. Indeed, all my fears were right. My new collaborator had systematically been committing fraud, replicating manuscripts using the same data and publishing by himself using my very ideas and sentences.

I confronted him; I wanted to receive an explanation, a reason for these actions. I copied all other co-authors in these communications. The three manuscripts had built international collaboration, and other parties had actively participated, and now we all were compromised. The first reaction received was that he was not aware that was an illegal action, and then, silence. No satisfactory answer was ever received, and more importantly, it seemed some of the other co-authors did not care, nor were surprised.

## The aftermath of deception

In the next coming days, I redacted several email letters describing the misconduct situation to the different journal’s editors, preprint services and especially to the main affiliations of this fraudulent person. The two manuscripts were withdrawn from the respective journals right away. Together with the third manuscript, none of the documents will ever be published. There is a long history and documentation showing that withdraws and retractions of scientific manuscripts may be the most relevant form of silently reporting scientific misconduct [2, 3], and now I was part of it. Editors from the journals and editorial houses where the duplicated documents had been published responded to investigate the case. However, after several months of waiting, and despite the multiple complain letters providing all the evidence to prove the misconduct act, no official sanctions have been taken by any of the journals and the documents remain still available online. Editors have the responsibility to pursue scientific misconduct in submitted or published manuscripts; however, editors are not responsible for conducting investigation or deciding whether a scientific misconduct occurred [4].

The preprint services response was very clear and conclusive, regardless of the evidence provided, the documents published online in their preprint format cannot and will not be removed. Now our names will remain associated with this person to posterity, another wonderful discovery. Release of early results in the format of preprints without going through the process of *peer-review* is an old well known issue of concern [5–7]. For the last few years I have been in favour and accepting the early release of preprint publications, this new experience has made me reconsider and change entirely this position. I find unacceptable that in spite of providing all evidence of research misconduct, fraud and duplication of data especially, a retraction of a preprint document is not possible for most preprint services available.

As for the consequences or sanctions imposed on this “researcher” by his own affiliate institutions, it also remains unknown as no reply or answer has been received until now. Additionally, some of his personal collaborators also included as co-authors during the editing process of the manuscripts, as it was claimed they “intellectually contributed” to the study, contacted me during the first weeks after withdrawing. These collaborators were unhappy about the decision taken, and complained asking: ‘‘*what is it really necessary to retract the documents entirely, in particular one manuscript already accepted and a second one in-review? Why was not this decision put into a vote among the co-authors?”* They did not considered to be an enough reason for withdrawing and claimed, “*It had been a rush and wrong decision”*. The answer was simple, it was a clear research misconduct act and the data has been duplicated and misused, my decision could not be clouded by the grief of losing three publications. Besides, I was the last author and corresponding author for all three manuscripts, and thus, the responsibility and final decision relied on me. Furthermore, and as a curious additional detail, all editors associated to the journals where the two-duplicated manuscripts were published, all are as well from the same region as this person. All these facts together allow me to reach the conclusion that misconduct practices may be relatively more common in some other parts of the world, and the research culture may play an important role in this type of practices, but we are still afraid to discuss about it [8]. There are no rigorous or systematic controls to regulate that one unique person can manipulate, duplicate with slight modifications in the text, and publish the same datasets in different journals, especially if the time between submissions is minimal. There are thousands of journals with many more thousands of editors in an infinite number of online platforms. Decisions over whether to retract or modify a study are more likely to take years than months, this time could potentially harmfully misinform [9] and damage the reputation of researchers [3] if any sanction is taken at all by the end [10]. Based on the previous rationale, this author who duplicated our work and published by himself may simply get away with it, two fraudulent copy/paste extra publications and zero consequences.

Hundreds of hour’s work and nearly a year of effort were lost in an instant. As many others, I believe I work and interact with researchers sharing similar values of honesty, openness and accountability pursuing to establish as an independent researcher to produce good science work. Yet every aspect of science, from the framing of a research idea to the publication of a manuscript, is susceptible to influences that can lead to misconduct [11]. By withdrawing at once three manuscripts, now associated to misconduct practices, my research colleagues and I will suffer the consequences of the current academia culture of “publish or perish” [12].

## Recommendations to avoid unpleasant research events

With two official retractions across the editorial offices of two major journals and three preprint documents that I cannot rig out, all associated to fraud and scientific misconduct; I am probably the less qualified person with the least authority to provide any feedback and even less, a short list of recommendations to prevent misconduct in research. Nevertheless, here I am. There are many general guidelines and basic rules to prevent, avoid and report misconduct actions [3, 13–15], the interested readers can get more information below in the reference list if they want to explore deeper into this. Using these guidelines as the main backbone, a short list of three main recommendations is presented in the lines below.

The first and possibly most important recommendation, despite the previous shared experience; always welcome collaboration after a well-throughout background check. This may sound contradictory, but contemporary science is based on collaboration and the interdisciplinary combination of fields [16], one bad experience and one “rotten apple” cannot disrupt the development of scientific research. Of course, it is mandatory to be vigilant and to carefully investigate the background interests [9] and history of each new door that opens along the way. Welcome collaboration cautiously.

A second recommendation, to investigate the institution and location of the new coming collaborations. As stated above, the cultural background [8], and thus, the location of these new collaboration institutions may play a very important role in the final outcome. Most countries across Europe and in the U.S. have well-defined guidelines [3, 10], which varied a lot about each principle and at the end are regulated by each institution research policies. However, there may be regions across the world where policies and regulations concerning misconduct actions and the implications and consequences are yet not well established [17]. Avoid those.

My third recommendation, and possibly the most relevant of all, do not take for granted that the other researchers are fully aware that some actions may lead to misconduct. My biggest mistake was to believe that other researchers knew or cared about the basic rules of duplication of data, transparency and respect of authorship rights. Ignorance still accounts for a large portion of the research misconduct actions [11, 18]. Never assume that others know and respect the broad spectrum of misconduct actions.

Two additional personal recommendations. Stay away from review manuscripts and book chapters, avoid them at all cost. Consider very carefully sharing your manuscript results in the format of an early release preprint online publication.

## Conclusions

There is so much to modify in the existing science research environment to avoid situations like this to continue or ever happen again. Young scientists need to be inspired and motivated to produce by example based on principles of integrity, ethical values, transparency and respect, and not by current trend of rejection and extreme pressure. Dealing with the research pressure to secure external funds and to publish in top-tier journals stand as the most common stressors that contribute to research misconduct [15, 19]. The same research culture that creates this pressure for publishing and obtaining funds, it also contributes to the behaviour practice of silence that leads to ignore and avoid the topic of misconduct in research. While there is a general concern and scientific journals attempt to take situations like this seriously, there should also be a more open space to share and inform junior and even senior researchers about this kind of predatory stealing research practices.

Manipulation and duplication of data to inflate academic records is a desperate and shameless act, and it truly represents scientific misconduct and fraud. Unfortunately, there is a general trend with an increase in misconduct in research [13], which ultimately account for the majority of withdrawals in modern scientific publications [20]. I would like to believe that even good people could do bad things when extreme pressure is received. Nevertheless, would this justify misconduct and fraud? Never!

## Data Availability

Not applicable.
